# Sanders’ Strictures on the Use of Ergot

**Published:** 1848

**Authors:** John H. Sanders


### ARTICLE III.

*Strictures on the use of Ergot as a Parturient Agent.* Extracted from a paper read before the Indianapolis Medical Society July 1st, 1848. By JOHN H. SANDERS, M.D.

Various and contradictory opinions have been and still are entertained respecting ergot. Some have considered it a fungus, which has been named spermœdia-clavus; others again consider it as the grain of rye stimulated into diseased action by the presence of the sporidia of a fungus. This opinion, promulgated by Seveillé, has been sustained by Quekett. Similar statements are made by Phillipar and Smith, leaving but little if any doubt but that the spurred rye results from a disease of the grain, caused by the presence of a parasitical fungus, or, as others suppose, by a combination of morbific causes, such as heat and moisture, impairing or interrupting the healthy development of the grain in its incipient state.

Upwards of two hundred years ago we find mention made of it as a popular remedy in the hands of the midwives of France and Germany; and it was then considered as possessing powers capable of accelerating labor. Indeed, long before the profession paid any attention to it as a therapeutical agent, we find the midwives of olden times had been in the habit of giving it when, in their sage judgments, something was necessary to hasten or increase the pains of labor.

Admitting it, then, to possess these powers of inducing and increasing uterine contraction, and, sometimes, of hastening labor, it becomes a question of grave importance, whether it is ever needed, and under what peculiar circumstances or conditions it may, with impunity, be administered to the parturient female. The friends and advocates of the article admit its inapplicability in all cases anterior to the birth, where the following conditions do not obtain. The os uteri must be soft and yielding, and, if not dilated, at least dilatable; the bladder of waters previously ruptured; the head or breech presenting; the uterine efforts feeble and inefficient, and no opposing obstacle to a natural delivery. Under such circumstances, it is said, it may be administered with the happiest effects. I will not say that, under such conditions it cannot be given without manifest injury to the patient or hazard to the child. But I do say that all the cases which have come under my supervision for upwards of twenty years have been conducted to a favorable issue without it. And when I reflect upon the misery and mischief which have grown out of its administration, I regret that it was not suffered to sleep with the midwives of France and Germany. Happy would it have been for the cause of humanity, and honorable to the profession, if our own distinguished countryman, Dr. Sterns, of New York, had not called the attention of physicians to it. Still more recently, the influence exerted in favor of it by Dewees has greatly contributed to its free administration, and, it is to be feared, not to the credit of the profession or the interest of humanity. None will, I presume, be so reckless, or through ignorance attempt to give it, if the os uteri be hard, rigid, or unyielding, and where the bladder of waters has not given way, and where the strength or tone of the general system is in a healthy condition, or when any other part presents save the head or breech. These seem to be the conditions governing its administration, which, if strictly attended to, might not lead to such mischievous results as too often attends its exhibition. But I have said it has not been necessary nor has it been absolutely indicated in any case which has come under my supervision for upwards of twenty years, and although I have had numberless cases characterized by all those symptoms on which its safe administration depends, yet I have always succeeded without it. It may be said by others, however, that while I have succeeded without it, yet it follows not as a corollary, that its use should be interdicted.

What are the objections to its administration? The first is predicated upon a want of positive knowledge as to what causes the delay, whether it is from inertia or deficient uterine action, or from a great variety of causes over which time and rest might exercise the most salutary influence, and in due time accomplish that which at first seemed remotely promised. Another objection is the great suffering which it superinduces, causing pains of the most insupportable character, altogether different in their nature from the natural and healthy contractions which attend unassisted labor. The contractions induced by ergot assume more the character of tonic spasms than of the natural alternate action and relaxation. They are of much greater strength and of longer continuance; and every female to whom it has been given, so far as has come to my knowledge, has expressed her decided disapprobation of its effects, and a disinclination to taking it the second time. Indeed, to me it has ever seemed that its effects upon the uterus were to produce contractile rather than expulsive efforts, and hence arises danger of its rupturing or lacerating the womb, if not administered under the most favorable circumstances. Pains to promote labor should be contractile and expulsive, otherwise great suffering is experienced and labor not advanced. This is the character of the pains during the first stage of confinement, described by females as cutting or grinding pains. Another objection which may be urged against its use, is the great liability of its inducing uterine inflammation. I have known quite a number of cases follow its exhibition more especially in primipari labor, where no other good reason could be assigned. Such a result is not unreasonable, but comports with the soundest principles of pathology; for what else could be expected than that inflammation would follow when the uterine tissues had been inordinately excited and made to labor beyond their strength? A morbid sensibility is thus created, and it would be strange if serious mischief did not result. This objection is of great weight, and if no other existed, it would be a sufficient cause for its entire rejection.

Another very serious objection to its exhibition is founded upon the acknowledged fact that it frequently causes the death of the child, or, at least, children are frequently still-born after its administration to the mother. The following are the remarks of a most distinguished physician of Europe upon the fatal effects of ergot. "In America it is observed that so many children are still-born after the exhibition of that article, that it has led many practitioners in this country to discontinue its use altogether." The reason of its fatal effects is not of difficult explanation, when we consider that the contractions produced by it are spasmodic, and of such a character as to interrupt the circulation of the maternal blood in the uterus and placenta for a long time, and thus prevent the necessary changes from taking place in the fœtal blood.

Doctor Hosack, of New York, speaking of this article says: "The ergot has been called, in some of the books, from its effects in hastening labor, the pulvis adpartum; as regards the child, it may, with almost equal truth be denominated the pulvis admortum, for I believe its operation, when sufficient to expel the child, in cases where nature alone is unequal to the task, is to produce so violent a contraction of the womb as frequently very much to impede, if not totally to interrupt the circulation between the mother and child. Superadded to all this, is the great danger of doing violence, not alone to the gravid uterus and its contents, but likewise to the vaginal parieties, upon the healthy integrity of which mainly depends the retaining of the uterus in its normal position, as it is now most generally admitted that prolapsus and prosedencia uteri are caused or greatly aggravated by a relaxation of the vaginal sheath. Again, there is a morbid sensibility following its administration; the healthy secretions are interrupted or suppressed; a predisposition to inflammation created; and, too often, protracted recovery follows in the train. I am aware that the weight of popular authority is against me. But, gentlemen, what I know and that which I have seen, are of more importance to me than what I have read. And while a Ramsbotham, a Churchill, a Dewees, and a Meigs, with many others of equal fame, may commend it to the attention of the profession, permit one although his pretensions may be humble, to raise a warning voice against its use, as I consider it as incompatible with the soundest principles of pathology, and at war with the interests of humanity. Especially is it unnecessary in this country, where our females are generally blessed with good constitutions. If medical gentlemen cannot spare the time necessary for nature to accomplish the work, they would do well to retire and leave their patient in the hand of some discreet female friend, or send for some other physician. In so doing, much suffering and mischief would be avoided. But it may be asked, Do you not find many cases of lingering and protracted labor, arising from inertia or a want of vigorous uterine contraction, in which, from all appearances, labor might be terminated by a few pains? I answer, unhesitatingly, in the affirmative. But there are other appliances or aids which are always at command, and to which I have resorted for many years, in such cases. First, if the patient be in that condition upon which the safe administration of the article depends, I would enjoin rest, and give encouraging assurances. And in the absence of fever, I should not hesitate to give a glass of wine or some other grateful and moderately stimulating cordial. If the bowels should be constipated, I would order an enema of molasses and water, or simply tepid water. Such means, although simple, very frequently awaken, or excite parturient pains amply sufficient to accomplish the birth of the child. If these means should fail, and if danger is not apprehended by delay, why resort to a doubtful to say the least, if not a dangerous remedy? I have had patients for hours and even days after the membranes had given way, and that, too, not prematurely, pass the time quite pleasantly, save a little mental disquietude, and yet do well ultimately. But should the importunities of your patient or the solicitude of friends seem to demand something more than the employment of the means above named, others might be tried, such as friction with the hand over the region of the uterus, or moderate pressure upon the abdominal muscles, or the application of cold wet cloths, or douches of cold water immediately over the hypogastric region. This practice, if adopted, will, in most cases, excite uterine contraction. In order to sustain and support that viscus, a binder or roller may then be applied moderately tight around the abdomen, in order to compress the abdominal muscles, and cause them to contract and co-operate in the great work of delivery.

Another objection to the use of ergot is this—that while all admit it to be an excitant and capable of producing violent parturient efforts or pains, its operation is exclusively confined to the uterine tissues and therefore this organ is made to labor inordinately. This is not the case with those means or instrumentalities which I have recommended to be brought into requisition. They not only excite uterine contractions, but, at the same time, bring to its aid the corresponding and co-operating efforts of the diaphragm and abdominal muscles, which is of great importance both during the period of the child's transit and afterwards as a means of preventing hæmorrhage, which frequently jeopardizes the life of the patient.

Permit me further to remark, that I have known ergot given by physicians and midwives to patients in the condition fully to justify its administration, as they have declared to me, when, to their surprise, it has seemed to counteract or subvert the order of labor, and instead of hastening the birth of the child, absolutely to arrest its further progress. This it does by exciting a spasmodic action or stricture in the circular bands or fibres of the cervix uteri; and frequently it becomes necessary to give some powerful anodyne for the purpose of overcoming or relaxing the rigidity consequent upon its use.

These, gentlemen, are a few of the objections I have to to its administration when given purposely and solely to expedite labor. I will now proceed to give my objections to its use, after the birth of the child, in order to hasten the expulsion of the placenta. And here let me ask, what is the condition or state of a female immediately subsequent to the birth of her child? Most generally it is that of extreme exhaustion, occasioned by the expenditure of power consequent upon the long and repeated efforts of nature to accomplish delivery. It is, therefore, necessary to give time and rest, and some restorative to favor the renewal of those healthy contractile efforts of the uterus which will generally follow in fifteen or twenty minutes after delivery. Should that, however, not be the case, and even should there be but little if any disposition in the uterus to close down upon itself, there is no danger in delay, and I have always found that, by having recourse to means above suggested, in due time, the object has been accomplished. Should there be hemorrhage from a partial separation of the placenta without due contraction, I should not delay, but bring immediately into requisition these means I have named, such as pressure, cold applications, &c., and even the immediate introduction of the hand into the uterus, which, not unfrequently, by its presence simply, brings about contraction. But in after hemorrhages, or hemorrhages occurring after the expulsion of the membranes, if friction, pressure, cold applications, &c., should not arrest it, I should then not hesitate to give ergot in sufficient doses to make a decided impression on the uterine fibres.

For the benefit of those of limited experience in the profession, I will here make (to my mind a practical) suggestion. Should you be called to attend a lady who had previously borne children, and who had generally suffered inconvenience after the birth of the child, either from retention of the placenta or hemorrhage, I would advise you to adjust the binder or roller around the abdomen, not so tight as to occasion inconvenience; and as labor advanced have it tightened, and, at the same time, keep back or hinder the immediate birth of the child, by gently pressing with the hand on the perinœum, in order to give the uterus time to contract upon itself as the child is expelled.

Another difficulty to the inexperienced may here present itself. Not unfrequently the placenta is retained after separtion, by an over or excessive contractile disposition of the uterus. This contraction may sometimes require bleeding to overcome it, and then, by the introduction of one or more fingers, with gentle traction at the cord, you will succeed. Should the placenta not be detached after you have freely bled your patient, the hand must be gradually insinuated into the uterine cavity and by the finger you may gently separate it, then, by the withdrawal of the hand it will follow. I have not unfrequently had to give tartar, ipecac., and opium, in addition to bleeding before I could overcome the contraction. But I find, gentlemen, I am extending my remarks beyond the limits contemplated at the beginning of this address, and shall therefore dismiss the subject, hoping that some practical good may result from this imperfect sketch, especially to the junior members of the profession.

